# Supplementary figures and images for: Association between sarcopenia and falls in Chinese older adults: Findings from the China health and retirement longitudinal study
Source: PLoS One. 2025 Jun 12;20(6):e0326193. doi: 10.1371/journal.pone.0326193 (PMC12161576; doi:10.1371/journal.pone.0326193)

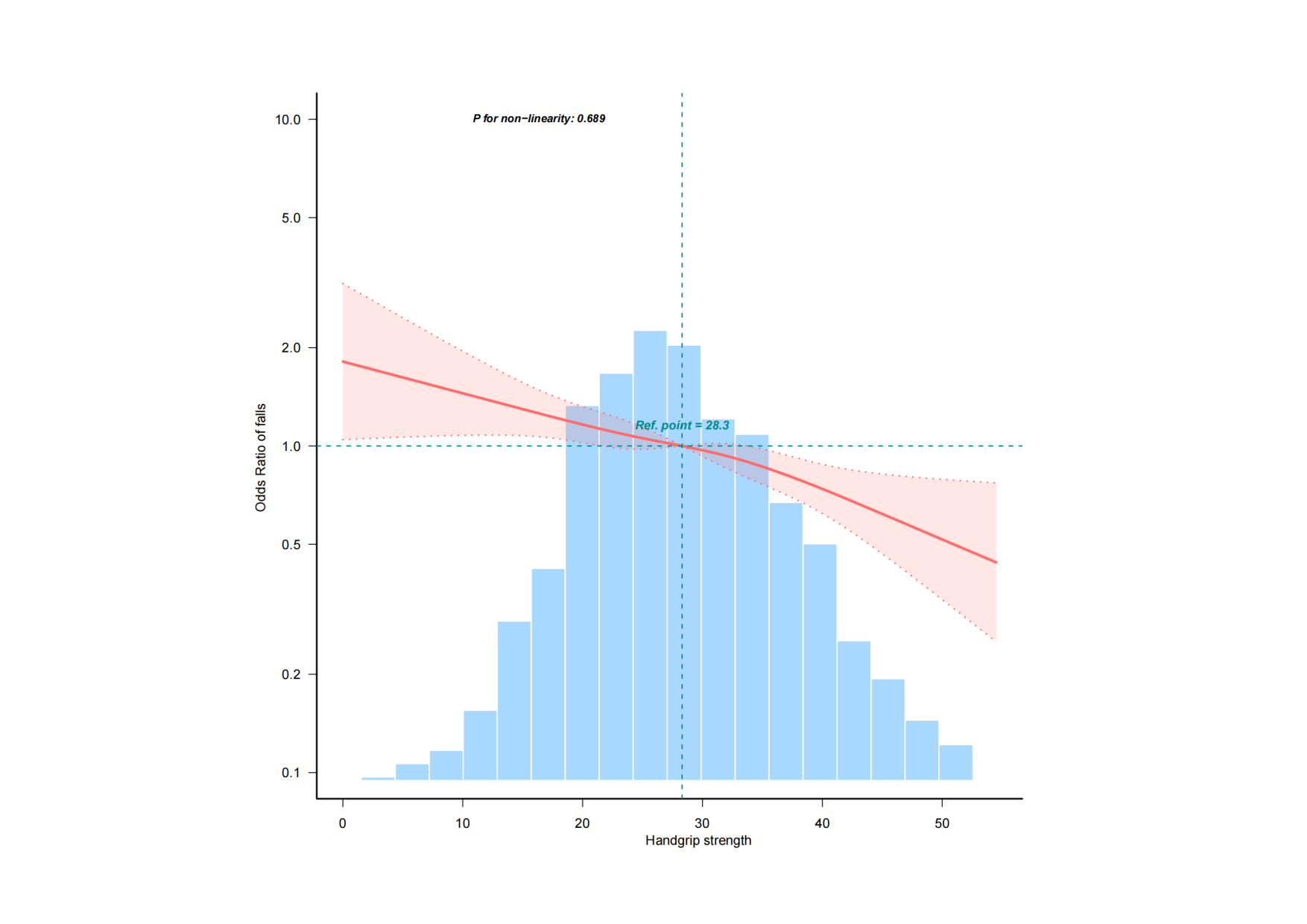

Supplement: S1 Fig — (TIFF) [file pone.0326193.s001.tif]

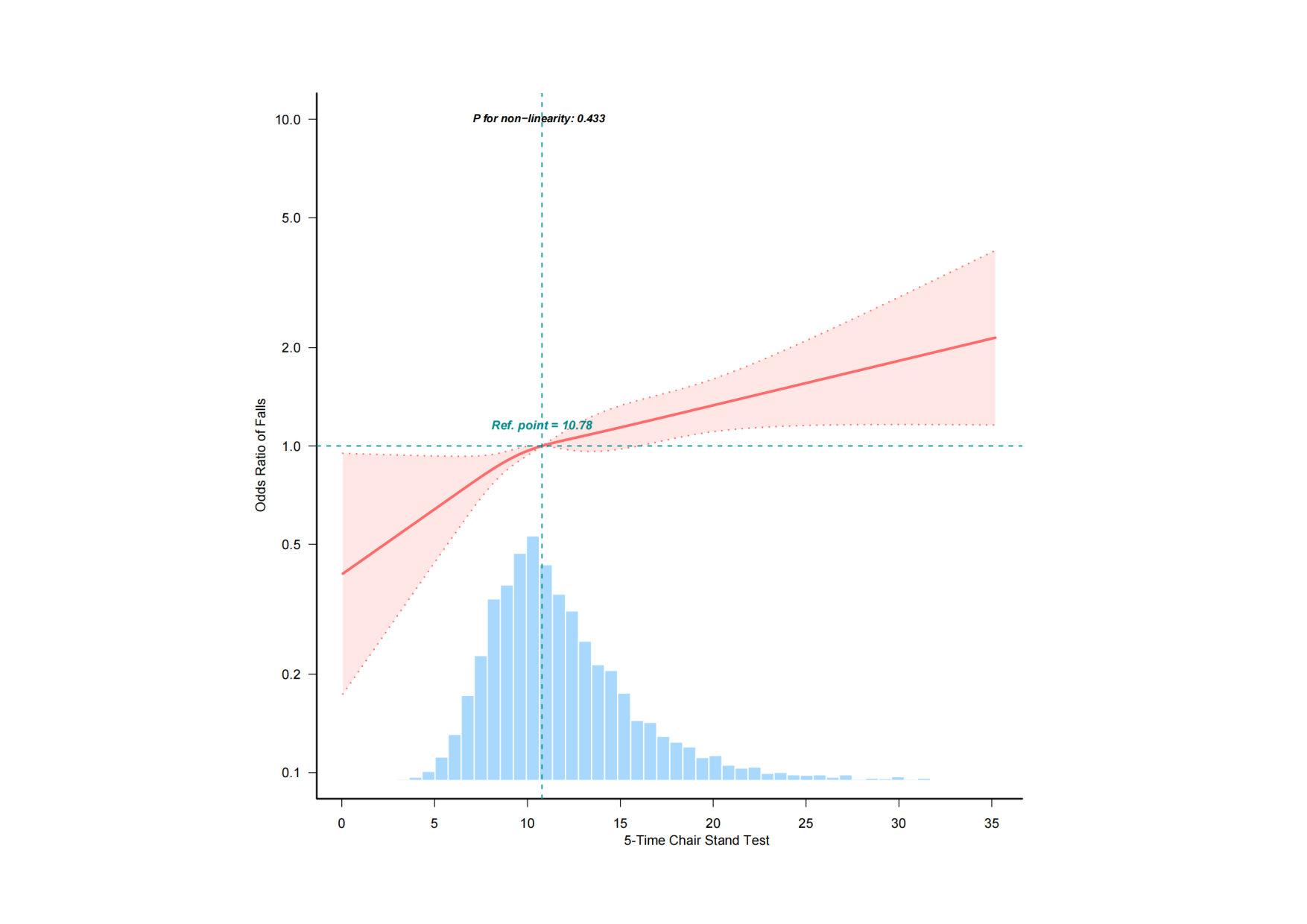

Supplement: S2 Fig — (TIFF) [file pone.0326193.s002.tif]
