# Supplementary material for: Association between sarcopenia and falls in Chinese older adults: Findings from the China health and retirement longitudinal study
Source: PLoS One. 2025 Jun 12;20(6):e0326193. doi: 10.1371/journal.pone.0326193 (PMC12161576; doi:10.1371/journal.pone.0326193)
Supplement: S1 Table — (DOCX) [file pone.0326193.s003.docx]

**Additional file**

**S1 Table.** **Cross-sectional association between components of sarcopenia and falls in 2011**

| **Sarcopenia**  **component** | **Crude model** | | **Model1** | | **Model2** | | **Model3** | |
| --- | --- | --- | --- | --- | --- | --- | --- | --- |
|  | **OR**  **(95%CI)** | p **value** | **OR**  **(95%CI)** | p **value** | **OR**  **(95%CI)** | p **value** | **OR**  **(95%CI)** | p **value** |
| Low muscle mass | 1.17 (1.01~1.37) | 0.042 | 1.09 (0.93~1.29) | 0.294 | 1.12 (0.94~1.33) | 0.215 | 1.08 (0.91~1.29) | 0.381 |
| Low handgrip strength | 1.47 (1.25~1.73) | <0.001 | 1.42 (1.2~1.67) | <0.001 | 1.4 (1.18~1.67) | <0.001 | 1.39 (1.17~1.66) | <0.001 |
| Low physical performance | 1.39 (1.21~1.6) | <0.001 | 1.27 (1.1~1.47) | 0.001 | 1.2 (1.03~1.39) | 0.018 | 1.21 (1.04~1.4) | 0.015 |

Crude model: no other covariates were adjusted.

Model1: we adjusted age and sex.

Model2:we adjusted modle1+Marriage status,education level,BMI,smoke,drink.

Model3:we adjusted modle2+Hgb,HbA1C,TG,UA,Cr,CyC,Chronic disease（included to hypertension,hyperlipidemia,diabetes,pulmanary,heart disease,stroke,kidney,digest,arthritis）


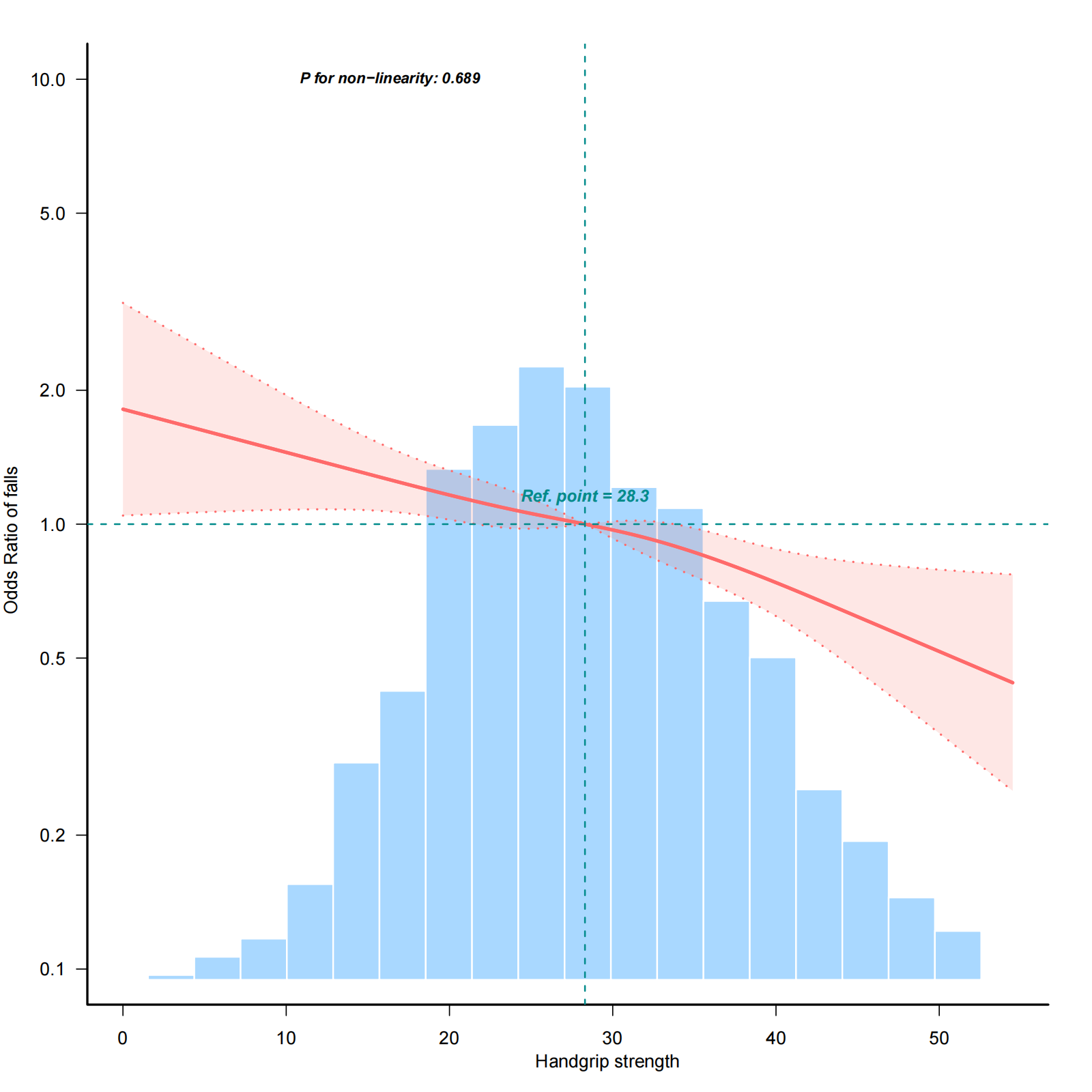


**Figure S1** Smooth curve ﬁtting of incident falls in older patients in CHARLS 2011 with handgrip strength. Solid and dashed lines represent the predicted value and 95% confidence intervals. They were adjusted for age, gender, Marriage status, education level, BMI, smoke, drink, Hgb,HbA1C,TG,UA,Cr,CyC,Chronic disease（included to hypertension, hyperlipidemia, diabetes, pulmanary diseases, heart disease, stroke, kidney diseases, digest tract disease, arthritis）. Only 99.8% of the data is shown.


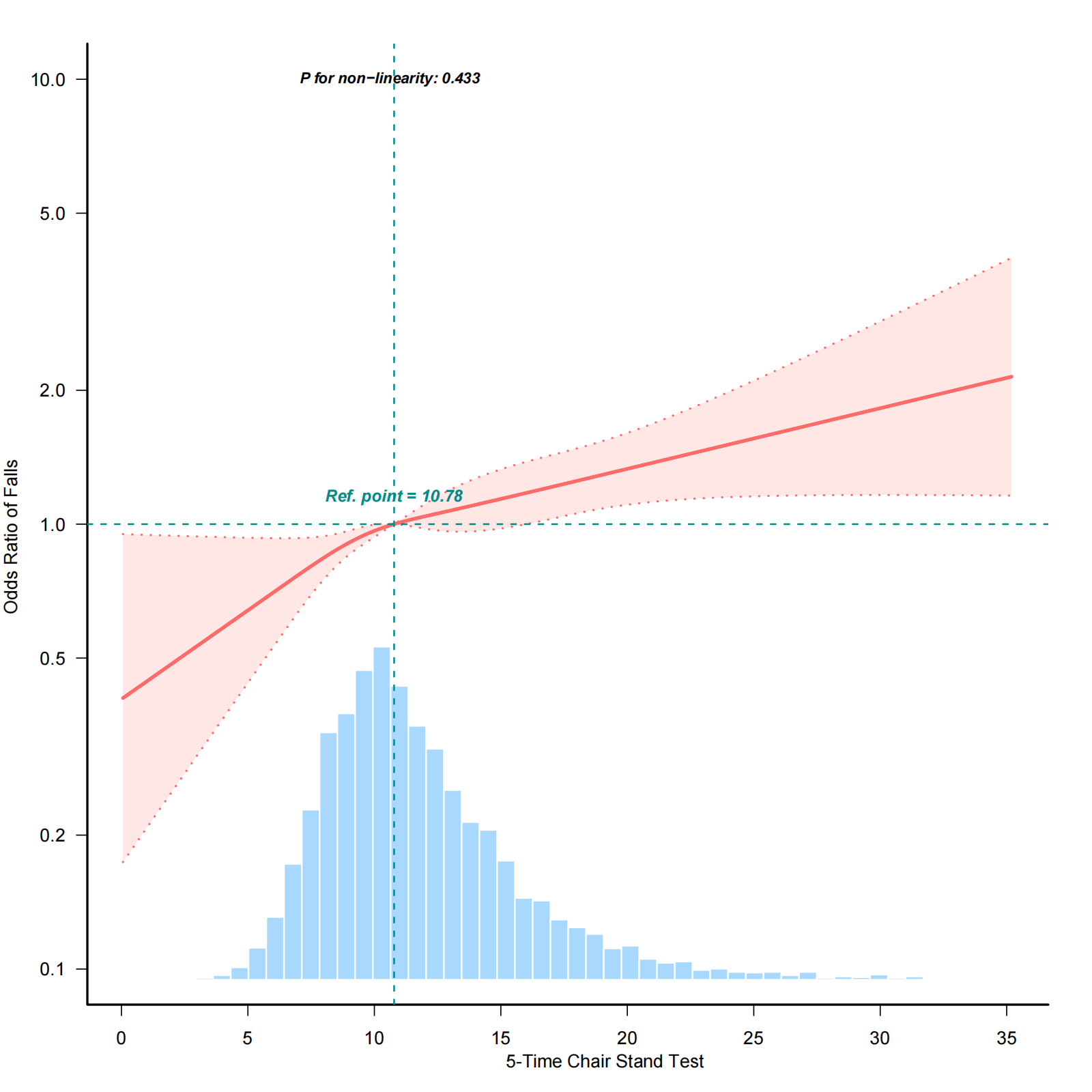


**Figure S2** Smooth curve ﬁtting of incident falls in older patients in CHARLS 2011 with 5-time chair stand test. Solid and dashed lines represent the predicted value and 95% confidence intervals. They were adjusted for age, gender, Marriage status, education level, BMI, smoke, drink, Hgb,HbA1C,TG,UA,Cr,CyC,Chronic disease（included to hypertension, hyperlipidemia, diabetes, pulmanary diseases, heart disease, stroke, kidney diseases, digest tract disease, arthritis）. Only 99.8% of the data is shown.
