# Supplementary material for: Association between sarcopenia and falls in Chinese older adults: Findings from the China health and retirement longitudinal study
Source: PLoS One. 2025 Jun 12;20(6):e0326193. doi: 10.1371/journal.pone.0326193 (PMC12161576; doi:10.1371/journal.pone.0326193)
Supplement: S2 Table — (DOCX) [file pone.0326193.s004.docx]

**Additional file**

**S2 Table.** **Longitudinal association between components of sarcopenia and falls , 2011–2015**

| **Sarcopenia**  **component** | **Crude model** | | **Model1** | | **Model2** | | **Model3** | |
| --- | --- | --- | --- | --- | --- | --- | --- | --- |
|  | **HR**  **(95%CI)** | p **value** | **HR**  **(95%CI)** | p **value** | **HR**  **(95%CI)** | p **value** | **HR**  **(95%CI)** | p **value** |
| Low muscle mass | 1.15 (1.01~1.29) | 0.029 | 1.1 (0.97~1.24) | 0.136 | 1.09 (0.97~1.24) | 0.158 | 1.11 (0.98~1.26) | 0.106 |
| Low handgrip strength | 1.3 (1.15~1.48) | <0.001 | 1.27 (1.12~1.45) | <0.001 | 1.26 (1.11~1.43) | <0.001 | 1.28 (1.12~1.46) | <0.001 |
| Low physical performance | 1.14 (1.02~1.27) | 0.023 | 1.12 (1~1.25) | 0.047 | 1.12 (1~1.25) | 0.044 | 1.14 (1.02~1.28) | 0.02 |

Crude model: no other covariates were adjusted.

Model1: we adjusted age and sex.

Model2:we adjusted modle1+Marriage status,education level,BMI,smoke,drink.

Model3:we adjusted modle2+Hgb,HbA1C,TG,UA,Cr,CyC,Chronic disease（included to hypertension,hyperlipidemia,diabetes,pulmanary,heart disease,stroke,kidney,digest,arthritis）
