# Supplementary material for: Association between sarcopenia and falls in Chinese older adults: Findings from the China health and retirement longitudinal study
Source: PLoS One. 2025 Jun 12;20(6):e0326193. doi: 10.1371/journal.pone.0326193 (PMC12161576; doi:10.1371/journal.pone.0326193)
Supplement: Attachment — Submitted filename: Response to Reviewers.docx [file pone.0326193.s006.docx]

**ID:** PONE-D-25-00109

**Title of Manuscript:** Association between sarcopenia and falls in Chinese older Adults: Findings from the China Health and Retirement Longitudinal Study

Dear Editors and Reviewers:

On behalf of my co-authors, we greatly appreciate the careful review and comments from both you and the reviewers. We believe that by implementing the suggested changes, we now have a stronger manuscript entitled “**Association between sarcopenia and falls in Chinese older Adults: Findings from the China Health and Retirement Longitudinal Study” (ID: PONE-D-25-00109)** for submission to PLOS ONE.

We have thoroughly reviewed the comments and implemented the necessary revisions, which we trust will meet with your approval. The revised sections are highlighted in red within the updated manuscript. These modifications do not affect the overall content or structure of the paper. Below, we have provided detailed point-by-point responses to each of the editor's and reviewers' comments, along with the corresponding revisions made to the manuscript. A comprehensive summary of the corrections and our responses to the feedback can be found in the **Revision Report**.

There are no conflicts of interest regarding this work. All authors have read the revised manuscript and approved its submission to PLOS ONE. Please do not hesitate to contact us if we can be of any further assistance. Thank you and best regards.

Yours Sincerely

Chunhua Yang

March 19, 2025

1 Jinzhou Medical University Graduate Training Base, the 960th Hospital of People’s Liberation Army Joint Logistics Support Force, Jinzhou, Liaoning, China

2 Department of Geriatrics, Shanghai Jiao Tong University Medical School Affiliated Ruijin Hospital Luwan Branch, Shanghai, China

**Revision Report**

First of all, I would like to express our sincere gratitude to the reviewers for their comments. These comments are all valuable and helpful for revising and improving our manuscript, as well as the important guiding significance to our researches. Based on suggestions, we have clarified the methodology, expanded the references, and strengthened the comparison with previous studies in the discussion. Additionally, we have refined ambiguous language and terminology in the manuscript to ensure clearer and more precise expression of our viewpoints. We have studied comments carefully and have made correction which we hope meet with approval. The revised sections in the manuscript are clearly highlighted in red text for easy reference. The summary of corrections and the responses to the editor's and reviewers' comments are listed below.

**Responds to editors:**

**Comment 1:**

Please ensure that your manuscript meets PLOS ONE's style requirements, including those for file naming. The PLOS ONE style templates can be found at

https://journals.plos.org/plosone/s/file?id=wjVg/PLOSOne_formatting_sample_main_body.pdf and

https://journals.plos.org/plosone/s/file?id=ba62/PLOSOne_formatting_sample_title_authors_affiliations.pdf

**Response 1:**

Thank you for your comment. Following a detailed examination of the manuscript, we noticed that the file naming conventions for several figures did not adhere to the journal's style guidelines. We have now revised these to ensure full compliance with PLOS ONE's formatting standards.The revisions are extensive and not listed individually. Modified sections in the manuscript are highlighted in red for easy reference.

**Comment 2:**

We noticed you have some minor occurrence of overlapping text with the following previous publication(s), which needs to be addressed:

①https://www.frontiersin.org/journals/medicine/articles/10.3389/fmed.2021.755705/full

②https://www.mdpi.com/2227-9067/8/12/1096

③https://pubmed.ncbi.nlm.nih.gov/36058563/

In your revision ensure you cite all your sources (including your own works), and quote or rephrase any duplicated text outside the methods section. Further consideration is dependent on these concerns being addressed.

**Response 2:**

Thank you for your constructive suggestion. Upon a further thorough review of the manuscript, we identified minor textual overlap between the " Assessment of Sarcopenia" section in the Methods and references ① and ③. The authors have revised this section accordingly and added references ① and ③ to appropriately attribute the content. However, upon reviewing the content, methods, and discussion sections, we found no overlap between reference ② and our manuscript. We kindly request the editor to provide specific guidance on this matter so that we can make the necessary revisions. (①=[20]，③=[18] )

*“Sarcopenia status was evaluated according to the AWGS 2019 algorithm, which incorporates three diagnostic components: muscle strength, appendicular skeletal muscle mass (ASM), and physical performance[13]. Muscle strength was assessed through handgrip strength measurement using a YuejianTM WL-1,000 dynamometer (Nantong Yuejian Physical Measurement Instrument Co., Ltd., Nantong, China)[1]. Participants performed maximal voluntary contractions with both dominant and non-dominant hands. Two measurements were taken for each hand with the dynamometer held at 90°flexion. The diagnostic thresholds for low grip strength were defined as < 28 kg for men and < 18 kg for women. Muscle mass was estimated by ASM using a validated anthropometric equation in Chinese residents[16,17], showing strong agreement with dual X-ray absorptiometry (DXA)[16,17]. ASM for the Chinese population was estimated using a physical measurement formula reported by a previous study[18]”*

Page 6, lines 104-114;

*“Due to the small sample size of the severe sarcopenia group (n=145, 2.7%), we combined it with the sarcopenia group for analysis to ensure statistical reliability, following established methodological precedents[20]. ”*

Page 7, lines 130-133;

References:

*“[18] Hu Y, Peng W, Ren R, Wang Y, Wang G. Sarcopenia and mild cognitive impairment among elderly adults: The first longitudinal evidence from CHARLS. J Cachexia Sarcopenia Muscle. 2022 Dec;13(6):2944–52. https://doi.org/ 10.1002/jcsm.13081 PMID: 36058563”*

Page 25, lines 464-466;

*“[20] Gao K, Ma WZ, Huck S, Li BL, Zhang L, Zhu J, et al. Association between sarcopenia and depressive symptoms in chinese older adults: evidence from the China health and retirement longitudinal study. Front Med. 2021;8:755705. https://doi.org/10.3389/fmed.2021.755705 PMID:34869454.”*

Page 25-26, lines 471-474;

**Comment 3:**

Thank you for stating the following financial disclosure:

“This research was supported by the 2023 PLA General Logistics Department Health Care Special Project [grant number 23BJZ45] from Yan Gao and the 2023 Shanghai Huangpu District Research Project [grant number HLM202202] from Chunhua Yang.”

Please state what role the funders took in the study.  If the funders had no role, please state: "The funders had no role in study design, data collection and analysis, decision to publish, or preparation of the manuscript." If this statement is not correct you must amend it as needed.

Please include this amended Role of Funder statement in your cover letter; we will change the online submission form on your behalf.

Response 3:

The original funding was provided by two individuals; however, following discussions, it has now been revised to a single funder, namely Gao Yan, with the funding information as follows: " the 2023 PLA General Logistics Department Health Care Special Project [grant number 23BJZ45] from Yan Gao." We have specified the role of this funder as *"methodology, supervision, funding acquisition, and writing—review & editing,"* and have added this information to the final section of the cover letter.

*“This research was supported by the 2023 PLA General Logistics Department Health Care Special Project [grant number 23BJZ45] from Yan Gao*

*Role of Funder statement:*

*Yan Gao is the corresponding author who also undertakes the work of methodology, supervision, funding acquisition, and writing—review & editing in the research.”*

the final section of the cover letter

**Responds to the reviewer’s comments:**

**Reviewer #1**

**Comment 1:**

Dear author, after carefully reading your manuscript, I congratulate you on your work. I have no comments to make. It is well developed, with a clear introduction and objectives, and a robust and replicable methodology. Thank you for reviewing your manuscript.

**Response 1:**

Thank you for your thorough evaluation and positive feedback on our manuscript. We are particularly grateful for the recognition of our work's clarity in introduction and objectives, as well as the robustness and replicability of our methodology. Our study represents the first large-scale longitudinal investigation of the sarcopenia-falls relationship in Chinese older adults using nationally representative data from CHARLS. The innovative application of the Asian Working Group for Sarcopenia criteria in this population provides a culturally relevant framework for assessing sarcopenia, while our robust statistical approach ensures strong reproducibility of findings across different aging populations. Most importantly, our findings provide practical implications for fall prevention strategies in elderly care, particularly through early identification and intervention of sarcopenia. We are encouraged by your supportive comments and believe this work will make a meaningful contribution to geriatric research in China. Once again, we deeply appreciate your time and valuable insights.

**Reviewer #2**

**Comment 1:**

This study investigates the correlation between sarcopenia and falls among older adults in China using data from the 2011 baseline and 2015 follow-up surveys of the China Health and Retirement Longitudinal Study (CHARLS). The authors employ cross-sectional and Cox proportional hazards regression analyses to identify a statistically significant increase in fall prevalence among individuals with sarcopenia compared to those without. This research makes a valuable contribution by emphasizing the importance of early screening and intervention for sarcopenia to reduce incidences of falls.

The study addresses a critical public health issue with a robust dataset, providing meaningful insight into the relationship between sarcopenia and falls in an aging population. The manuscript is well-structured and delivers a thorough statistical analysis, crucial for understanding the dynamics between sarcopenia and fall risk. However, there are several issues that limit the study's value and raise concerns.

**Response 1:**

We sincerely appreciate your insightful comments and positive evaluation of our work. We are particularly grateful for the recognition of our study's contribution to understanding the relationship between sarcopenia and falls in China's aging population. Acknowledging our methodological approach, including using CHARLS data and robust statistical analyses, is especially encouraging. We agree that early sarcopenia screening could significantly impact fall prevention strategies, and we thank you for highlighting this important implication of our findings. We sincerely appreciate the reviewer's valuable comments and will address each point to enhance the study's rigor and impact.

**Comment 2:**

A significant limitation, as explicitly reported in the manuscript, is the use of anthropometric equations to estimate muscle mass. This method is not as precise as imaging techniques such as DXA or BIA, potentially affecting the accuracy of sarcopenia diagnoses.

**Response 2:**

Although our study utilized anthropometric equations rather than the DXA or BIA techniques recommended by AWGS 2019, substantial evidence supports the validity of this approach. Previous studies have demonstrated strong concordance between ASM estimated by anthropometric prediction equations and DXA measurements through cross-validation. When DXA scanning is unavailable or impractical, anthropometric prediction equations serve as a reliable alternative for assessing appendicular skeletal muscle mass without compromising the diagnostic accuracy of sarcopenia. We have addressed this methodological consideration in the "Strengths and Limitations" section of our manuscript and have incorporated additional supporting references [46,47] to strengthen our methodological justification.

*“Second, while our study used anthropometric equations to assess muscle mass instead of AWGS 2019 recommended DXA/BIA, this method has been validated for the Chinese population. Studies show strong agreement between ASM estimated by anthropometric equations and DXA measurements[46,47]. Although less precise than imaging, it provides a practical, reliable alternative for large-scale studies like CHARLS, balancing accuracy and feasibility. ”*

Page 21, lines 373-377

References:

*“[46] Villani AM, Crotty M, Cameron ID, Kurrle SE, Skuza PP, Cleland LG, et al. Appendicular skeletal muscle in hospitalised hip-fracture patients: development and cross-validation of anthropometric prediction equations against dual-energy X-ray absorptiometry. Age Ageing. 2014 Nov;43(6):857–62. https://doi.org/10.1093/ageing/afu106 PMID: 25049262*

*[47]Wen X, Wang M, Jiang CM, Zhang YM. Anthropometric equation for estimation of appendicular skeletal muscle mass in chinese adults. Asia Pac J Clin Nutr. 2011;20(4):551–6.https://doi.org/10.6133/apjcn.2011.20.4.13 PMID: 22094840 ”*

Page 30, lines 556-562;

**Comment 3:**

Additionally, the analysis relies on self-reported incidences of falls, which may introduce recall bias and affect the reliability of the results.

**Response 3:**

We sincerely appreciate the reviewer’s insightful comment regarding the potential for recall bias in self-reported fall data. We acknowledge that self-reported measures may introduce some degree of recall bias; however, we would like to highlight several points supporting the validity and reliability of our findings based on the self-reported fall data in this study.

Firstly, self-reported fall data have been widely used in large-scale epidemiological studies, particularly in aging populations, and their validity has been well-documented. Studies have shown that self-reported falls in older adults exhibit a high concordance with objective measures, such as accelerometer data, especially when collected through structured interviews. For example, Ganz et al. demonstrated that self-reported falls in community-dwelling older adults had a sensitivity of 70%-80% compared to objective measures, supporting their utility in large cohort studies [1]. Similarly, Lusardi et al. found that self-reported fall events were reliable and valid for identifying fall risk in older populations, particularly when collected through standardized questionnaires [2].

Secondly, The China Health and Retirement Longitudinal Study (CHARLS) employs rigorous data collection protocols, including face-to-face interviews and standardized questions, to minimize recall bias. The use of a binary response (“yes” or “no”) to the question “Have you had a fall in the past two years?” or “Have you experienced a fall since your last visit?” is a well-established method for capturing fall incidents in longitudinal studies. Additionally, CHARLS incorporates follow-up questions to specify the number of falls requiring medical treatment, which further enhances the accuracy of the data. These methodological strengths have been validated in previous studies utilizing CHARLS data [3].

Thirdly, While recall bias cannot be entirely eliminated, the longitudinal design of CHARLS, with biennial follow-ups, reduces the risk of long-term recall errors. Furthermore, the large sample size and population-based nature of CHARLS help mitigate the impact of individual recall variability on the overall results. The consistency of our findings with prior literature on sarcopenia and fall risk also supports the robustness of our conclusions.

In summary, while recall bias cannot be entirely ruled out, the methodological rigor of CHARLS and the established validity of self-reported fall data support the reliability of our conclusions.

*“First, variables such as falls and chronic diseases were self-reported, which may introduce recall bias. However, self-reported data on these outcomes are widely used and validated in aging studies. Rigorous quality control measures in CHARLS minimize reporting errors, ensuring the robustness of our findings despite potential biases.”*

Page :21 lines:369-373

References:

[1] Ganz DA, Latham NK. Prevention of Falls in Community-Dwelling Older Adults. N Engl J Med. 2020;382(8):734-743. doi:10.1056/NEJMcp1903252

[2] Lusardi MM, Fritz S, Middleton A, et al. Determining Risk of Falls in Community Dwelling Older Adults: A Systematic Review and Meta-Analysis Using Posttest Probability. J Geriatr Phys Ther. 2017;40(1):1-36. doi:10.1519/JPT.0000000000000099.

[3] Zhao Y, Hu Y, Smith JP, Strauss J, Yang G. Cohort Profile: The China Health and Retirement Longitudinal Study (CHARLS). Int J Epidemiol. 2014;43(1):61-68. doi:10.1093/ije/dys203

**Comment 4:**

Furthermore, it is uncertain whether these results are generalizable to non-Asian populations.

**Response 4：**

Thank you for your valuable comment regarding the generalizability of our findings to non-Asian populations. We agree that our study, conducted in a Chinese cohort, may have limitations in generalizing the results to other ethnic groups due to differences in genetics, lifestyle, and healthcare systems. However, evidence from other regions supports the idea that sarcopenia is a significant risk factor for falls across diverse populations. For example, Veronese et al.[1] conducted a study in five low- and middle-income countries, demonstrating that sarcopenia is strongly associated with fall-related injuries among older adults, regardless of geographic or cultural context. Similarly, Beaudart et al.[2]found consistent results in European populations, highlighting the global relevance of sarcopenia as a fall risk factor.

These studies suggest that the biological mechanisms linking sarcopenia to falls, such as muscle weakness and impaired balance, are likely universal. That said, we recognize that factors like physical activity levels, dietary habits, and access to healthcare may vary across populations and could influence the relationship between sarcopenia and falls. Therefore, while our findings are specific to Chinese older adults, they contribute to a broader understanding of sarcopenia as a global health issue. Your comment has inspired us to consider future research in more diverse populations to further validate and expand on these findings. We thank you for your constructive feedback, which offers valuable direction for future research, such as comparing the incidence of falls related to sarcopenia among elderly populations in Europe, Asia, and Africa.

References:

[1]Veronese N, Smith L, Barbagallo M, Yang L, Zou L, Haro JM, Koyanagi A. Sarcopenia and fall-related injury among older adults in five low- and middle-income countries. Exp Gerontol. 2021 May;147:111262. doi: 10.1016/j.exger.2021.111262,PMID: 33516908.

[2]Beaudart C, Zaaria M, Pasleau F, Reginster JY, Bruyère O. Prevention of Falls in Community-Dwelling Older Adults. PLoS One. 2017 Jan 17;12(1):e0169548. doi: 10.1371/journal.pone.0169548,PMCID: PMC5240970.

**Comment 5:**

The criteria for selecting variables for the multivariate analysis are also not well described.

**Response 5:**

Thank you for your valuable comment regarding the criteria for selecting variables in our multivariable analysis. We appreciate your suggestion to clarify this aspect of our methodology. In the original manuscript, we briefly mentioned the variable selection criteria in the “Statistical analysis”, stating: “Furthermore, the selection of confounders was based on clinical relevance, existing scientific literature, and all significant covariates identified in the univariate analysis.” However, we acknowledge that this description was somewhat general. Based on your feedback, we have revised this section to provide a clearer and more detailed explanation of our variable selection process. We believe these revisions have significantly improved the transparency and rigor of our methods.

*“Variables with a univariate association ( p < 0.10 ) or clinical relevance were included in multivariable logistic regression models. Three hierarchical models were constructed: Model 1 adjusted for age and sex; Model 2 added adjustments for marital status, education, BMI, smoking, and alcohol consumption; and Model 3 further adjusted for biochemical markers ( Hgb, HbA1c, TG, UA, Cr, CyC ) and chronic diseases.”*

Page :9 lines: 173-177

**Comment 6:**

It remains unclear whether an analysis of gait and gait disorders was conducted in these patients. In this regard, see and discuss “Tinetti mobility test is related to muscle mass and strength in non-institutionalized elderly people. Age (Dordr). 2016 Dec;38(5-6):525-533”

**Response 6:**

Thank you for your insightful comment regarding the analysis of gait and gait disorders in our study. We appreciate your suggestion to consider the relationship between gait, muscle mass, and strength, as highlighted in the study by Cesari et al[1]. However, we would like to clarify that the CHARLS database does not include specific indicators related to gait or the Tinetti Mobility Test. Despite this limitation, our findings on the association between sarcopenia and falls remain robust, as sarcopenia itself is a well-established risk factor for functional decline and falls, independent of gait assessment.

Several studies have demonstrated that sarcopenia, characterized by low muscle mass and strength, is strongly associated with an increased risk of falls, even in the absence of direct gait measurements. For example, Landi et al[2] found that sarcopenia significantly predicted falls in older adults, emphasizing the importance of muscle quality and strength. Similarly, Yeung et al[3] reported that sarcopenia was independently associated with an increased risk of falls in community-dwelling older adults, further supporting our findings. While gait analysis could provide additional insights, the absence of such data in CHARLS does not diminish the validity of our findings. We believe that our study contributes valuable evidence on the relationship between sarcopenia and falls, supporting the need for early screening and intervention in older adults.

We have incorporated your valuable suggestion into the "Discussion" section of our manuscript (Lines 302-309), which has enriched the content of our discussion and provided important direction for our future research. Thank you again for your thoughtful comment, which has significantly improved the quality of our work.

*“Although the CHARLS database does not include specific indicators related to gait or gait disorders, such as the Tinetti Mobility Test, previous studies have highlighted the importance of gait and balance in fall risk assessment. For example, Cesari et al. demonstrated that the Tinetti Mobility Test is closely related to muscle mass and strength in non-institutionalized elderly individuals, suggesting that gait assessment could provide additional insights into fall risk[28]. While our study did not directly assess gait, the strong association between sarcopenia (a key determinant of muscle mass and strength) and falls further supports the importance of muscle-related factors in fall prevention.”*

Page :17-18 lines: 296-303

*“[28]Curcio F, Basile C, Liguori I, Della-Morte D, Gargiulo G, Galizia G, et al. Tinetti mobility test is related to muscle mass and strength in non-institutionalized elderly people. Age Dordr Neth. 2016;38(5–6):525–33. https://10.1007/s11357-016-9935-9 PMID:27566307”*

Page :27 lines: 499-501

References:

[1] Cesari M, Demougeot L, Boccalon H, et al. Tinetti mobility test is related to muscle mass and strength in non-institutionalized elderly people. Age, 38(5-6), 525-533.doi: 10.1007/s11357-016-9935-9. PMID: 27566307

[2] Landi F, Liperoti R, Russo A, et al. (2012). Sarcopenia as a risk factor for falls in elderly individuals: Results from the ilSIRENTE study. Journal of the American Medical Directors Association, 13(2), 121-126. doi: 10.1016/j.clnu.2012.02.007. PMID: 22414775.

[3] Yeung SSY, Reijnierse EM, Pham VK, Trappenburg MC, Lim WK, Meskers CGM, et al . Sarcopenia and its association with falls and fractures in older adults: A systematic review and meta-analysis. J Cachexia Sarcopenia Muscle. 2019;10(3):485-500. doi: 10.1002/jcsm.12411. PMID: 30993881

**Comment 7:**

The discussion lacks comparisons with two other studies using the same CHARLS dataset but missing the longitudinal analysis: one by Wu et al. which examines the prevalence and associated factors of sarcopenia “Sarcopenia prevalence and associated factors among older Chinese population: Findings from the China Health and Retirement Longitudinal Study. PLoS One. 2021 Mar 4;16(3):e0247617”, and another by Liang et al. which investigates the mediating role of sarcopenia in the association between physical activity and falls “The Mediating Role of Sarcopenia in the Association between Physical Activity and Falls among Chinese Older Adults: A Cross-Sectional Study. Healthcare (Basel). 2023 Dec 12;11(24):3146”. These comparisons could enrich the discussion by providing a broader context of sarcopenia research within the same population.

**Response 6:**

We greatly appreciate the reviewer's insightful suggestion. We have carefully addressed the comment by incorporating comparisons with the two studies mentioned (Wu et al.[39] and Liang et al. [40]) into the "Discussion" section of our manuscript (lines 343-353). These comparisons provide a broader context for sarcopenia research within the same CHARLS dataset population, highlighting consistencies and unique contributions of our longitudinal analysis. We believe this addition significantly enriches the discussion and strengthens the overall impact of our study.

*“To contextualize our findings within sarcopenia research using the CHARLS dataset, two relevant studies are noteworthy. Wu et al. reported a sarcopenia prevalence of 21.7% among older Chinese adults, highlighting factors such as age, gender, and physical activity levels[39], which align with our findings. Liang et al. demonstrated that sarcopenia mediates the relationship between physical activity and falls[40], complementing our results by elucidating the interplay between these factors in fall risk among older adults with sarcopenia.”*

Page :19 lines: 337-342

References:

*“[39] Wu X, Li X, Xu M, et al. Sarcopenia prevalence and associated factors among older Chinese population: Findings from the China Health and Retirement Longitudinal Study. PLoS One.2021;16(3):e0247617. https://doi.org/10.1371/journal.pone.0247617 PMID: 33661964*

*[40] Liang C, Shi L, Li B, He Z. The Mediating Role of Sarcopenia in the Association between Physical Activity and Falls among Chinese Older Adults: A Cross-Sectional Study. Healthc Basel Switz. 2023 Dec 12;11(24):3146. https://doi.org/10.3390/healthcare11243146 PMID: 38132036”*

Page :29 lines: 532-538

**Comment 7:**

Minor issues in the manuscript include occasional awkward phrasing and grammatical errors that could hinder comprehension. There are also inconsistencies in terminology, such as alternating between "probable sarcopenia" and "possible sarcopenia" without clear distinctions, potentially confusing readers. Addressing these linguistic and terminological issues would enhance the clarity and professionalism of the manuscript.

**Response 7:**

We sincerely thank the reviewer for pointing out these minor linguistic and terminological issues. We have carefully revised the manuscript to address the inconsistencies, such as standardizing the use of "possible sarcopenia" throughout the text to avoid confusion. Additionally, we have adjusted awkward phrasing and corrected grammatical errors to improve clarity and overall readability. The revised sections are highlighted in red within the text, but due to the extensive content, they are not listed here individually.These changes have been implemented to enhance the professionalism and coherence of the manuscript.

In conclusion, we sincerely appreciate the editor and reviewers' constructive comments and suggestions, which have significantly improved the quality of our manuscript. We have carefully addressed all the points raised and hope that the revised version meets the journal's standards. Thank you for your time and consideration.
