# Supplementary material for: Association between sarcopenia and falls in Chinese older adults: Findings from the China health and retirement longitudinal study
Source: PLoS One. 2025 Jun 12;20(6):e0326193. doi: 10.1371/journal.pone.0326193 (PMC12161576; doi:10.1371/journal.pone.0326193)
Supplement: Attachment — Submitted filename: 2Response_letter.docx [file pone.0326193.s007.docx]

**ID:** PONE-D-25-00109

**Title of Manuscript:** Association between sarcopenia and falls in Chinese older Adults: Findings from the China Health and Retirement Longitudinal Study

Dear editors：

Thank you for your constructive feedback and recognition of our revised manuscript titled "Association between Sarcopenia and Falls in Chinese Older Adults: Findings from the China Health and Retirement Longitudinal Study". We sincerely appreciate the time and effort invested by the editors and reviewers in evaluating our work.

1. Language Polishing

In response to the editorial advice, the manuscript has undergone comprehensive English language editing to enhance clarity and academic rigor. Key revisions include typographical corrections (e.g., "ealth" → "health" in Line 34 of the Abstract), restructuring of ambiguous sentences (such as the paragraph in the Conclusion section), standardization of terminology (e.g., replacing "older people" with "older adults" ), and formatting adjustments to align with PLOS ONE guidelines (e.g., units and punctuation). All modifications are highlighted in the "Revised Manuscript with Track Changes" file for transparency.

2. Reference Updates

In accordance with PLOS ONE’s data policy, we reviewed the reference list and replaced two citations:

1) Original Reference 2: United Nations. (2019). World population prospects 2019: highlights. United Nations.Retreved May 1, 2024, from: https://www.un-ilibrary.org/content/books/9789210042352

Updated Reference 2: Zhou M, Wang H, Zeng X, Yin P, Zhu J, Chen W, et al. Mortality, morbidity, and risk factors in China and its provinces, 1990-2017: a systematic analysis for the Global Burden of Disease Study 2017. Lancet. 2019;394(10204):1145-1158. doi: 10.1016/S0140-6736(19)30427-1 PMID: 31248666

(Line: 413-416)

2) Original Reference 47: Wen X, Wang M, Jiang CM, Zhang YM. Anthropometric equation for estimation of appendicular skeletal muscle mass in chinese adults. Asia Pac J Clin Nutr. 2011;20(4):551–6. https://doi.org/10.6133/apjcn.2011.20.4.13 PMID: 22094840

Updated Reference 47: Kawakami R, Miyachi M, Tanisawa K, Ito T, Usui C, Midorikawa T, et al. Development and validation of a simple anthropometric equation to predict appendicular skeletal muscle mass. Clin Nutr. 2021;40(11):5523-5530. doi: 10.1016/j.clnu.2021.09.032I PMID: 34656948. (Line: 559-561)

No retracted articles are cited in the revised manuscript.

Submitted Files

Response to Editor: Detailed responses to editorial comments.

Revised Manuscript with Track Changes: All modifications highlighted.

Manuscript (Clean Version): Final unmarked version.

Data Availability

All data are publicly accessible via the CHARLS repository (http://charls.pku.edu.cn), consistent with the Data Availability Statement.

Should further revisions be required, please do not hesitate to contact us. We look forward to your favorable decision.

Yours Sincerely

Chunhua Yang^1,2^

May 4, 2025

On behalf of all authors

1 Jinzhou Medical University Graduate Training Base, the 960th Hospital of People’s Liberation Army Joint Logistics Support Force, Jinzhou, Liaoning, China

2 Department of Geriatrics, Ruijin Hospital Lu Wan Branch, Shanghai Jiaotong University School of Medicine, Shanghai, China
